# The association between depressive symptoms in the community, non-psychiatric hospital admission and hospital outcomes: A systematic review

**DOI:** 10.1016/j.jpsychores.2014.11.002

**Published:** 2015-01

**Authors:** A. Matthew Prina, Theodore D. Cosco, Tom Dening, Aartjan Beekman, Carol Brayne, Martijn Huisman

**Affiliations:** a Department of Public Health & Primary Care, Institute of Public Health, Cambridge University, UK; b NIHR Collaboration for Leadership in Applied Health Research & Care for Cambridgeshire & Peterborough (CLAHRC-CP), UK; c Division of Psychiatry, Institute of Mental Health, University of Nottingham, UK; d Department of Epidemiology & Biostatistics, VU University Medical Center, Amsterdam, The Netherlands; e EMGO Institute for Health and Care Research, VU University Medical Center, Amsterdam, The Netherlands; f Department of Psychiatry, VU University Medical Center, Amsterdam, The Netherlands; g Department of Sociology, VU University, Amsterdam, The Netherlands; h King's College London, Institute of Psychiatry, Health Service and Population Research Department, Centre for Global Mental Health, London, UK

**Keywords:** Depression, Depressive symptoms, Hospitalisation, Comorbidity, Length of stay, Patient re-admission

## Abstract

**Objectives:**

This paper aims to systematically review observational studies that have analysed whether depressive symptoms in the community are associated with higher general hospital admissions, longer hospital stays and increased risk of re-admission.

**Methods:**

We identified prospective studies that looked at depressive symptoms in the community as a risk factor for non-psychiatric general hospital admissions, length of stay or risk of re-admission. The search was carried out on MEDLINE, PsycINFO, Cochrane Library Database, and followed up with contact with authors and scanning of reference lists.

**Results:**

Eleven studies fulfilled our inclusion and exclusion criteria, and all were deemed to be of moderate to high quality. Meta-analysis of seven studies with relevant data suggested that depressive symptoms may be a predictor of subsequent admission to a general hospital in unadjusted analyses (RR = 1.36, 95% CI: 1.28–1.44), but findings after adjustment for confounding variables were inconsistent. The narrative synthesis also reported depressive symptoms to be independently associated with longer length of stay, and higher re-admission risk.

**Conclusions:**

Depressive symptoms are associated with a higher risk of hospitalisation, longer length of stay and a higher re-admission risk. Some of these associations may be mediated by other factors, and should be explored in more details.

## Highlights

• We reviewed the relationship between depression and non-psychiatric hospitalisation.
• Depressive symptoms were associated with hospitalisation (RR = 1.4, 95% CI: 1.3–1.4).
• Adjustment for confounders reduced the magnitude of associations.
• Depression was also associated with longer length of stay and re-admission.

## Introduction

One of the most common mental disorders is depression. Although the prevalence of this disorder is reported to vary across countries and age groups [1], its global public health implications should not be underestimated. Depression is a primary cause of disability and functional limitations [2], reduced quality of life [3], and mortality [4]. Depression is also associated with a number of physical conditions, such as cancer, cardiovascular diseases and diabetes, among others [5–7].

Depressive symptomatology is extremely common among hospitalised patients, and this has led to increasing number of studies exploring the association between depressive symptoms and non-psychiatric hospital admission. The majority of the literature has however focused on clinical populations and individuals with pre-existing co-morbid conditions, rather than on community-dwelling people. This link has been highlighted in other reviews of clinical populations [8], where depression was reported to be associated with increased urgent healthcare use. It is not clear whether a relationship between depressive symptoms and hospitalisation may be the result of higher rates of physical illnesses in people with depressive symptoms, or whether this relationship may be independent of other factors. There is however some evidence that suggests that depression may be causally related to hospital admissions and outcomes, by influencing diverse pathways. Biological mechanisms, delayed access to care, poor treatment compliance, and direct influence on disability have all been described as potential mechanisms [9–12]. Studying the relationship between depressive symptoms and hospitalisation is important, because hospital care is costly and especially so when there are inefficiencies such as prolonged admissions. Health care costs are on the increase across the world [13] and understanding determinants of hospitalisation, length of stay and re-admission is a priority for policy makers, in particular as healthcare costs have been reported to be higher in people with depressive symptoms compared to those without it [14–17]. Evidence of a relationship between depressive symptoms and hospital outcomes would further highlight the need for prevention and adequate treatment of depression in the community, especially among otherwise frail and high-risk populations, and it may also suggest a stronger need for adequate screening for depressed mood in hospitalised population and for a role of liaison psychiatry.

In this review we have summarised the evidence on the relationship between depressed mood in community dwelling individuals, hospital admission and hospital outcomes. We think that this frame of enquiry is important as it looks at the impact of depression and patterns of hospital usage in total, and complements studies of the impact of single disorders. The outcomes that are researched are: non-psychiatric hospital admission, length of stay, and re-admission. We analyse both studies that have investigated depression/depressive symptoms as a risk indicator for more or longer hospital admissions, and those that aimed to unravel a causal relationship between depression and hospital admission (i.e. adjusted for potential confounders). Investigating the causal relationship between depression and hospital admission would provide a rationale for more timely or assertive treatment of depression, hoping to avert unnecessary hospitalisation, whereas investigating depression as an indicator of hospital admission is important in its own right, because it would allow the identification of high-risk individuals, and would be easily measured in day-to-day clinical practice.

### Aims of the study

This systematic review aims to provide a synthesis of the studies that have investigated the association between depressed mood or depressive symptoms, non-psychiatric hospital admission and hospital outcomes in the population as a whole. We aimed to evaluate the following research questions:

- Is depression or depressive symptoms associated with an increased risk of general hospital admission, for non-psychiatric causes? Is there still an association after adjustment for potential confounders?
- Is depression or depressive symptoms associated with other hospital outcomes (i.e. length of stay and re-admission) after adjustment for potential confounding variables?

## Method

### Data sources and searches

The methodology of our review followed the checklist proposed by the Meta-analysis of Observational Studies in Epidemiology (MOOSE) Group [18]. A literature search to explore the association between depression/depressive symptoms, hospitalisation and related outcomes was carried out. The search strategy included Medline, Ovid SP, PsycINFO and the Cochrane Collaboration database. An original search was carried out in October 2010, with further searches to update the results conducted in July 2012, and March 2014, with no time limits set. The search strategy conducted in Medline used medical subject headings (MeSH) applied in the following fashion: (“Hospitalization”[Mesh] OR “Patient Admission”[Mesh] OR “Patient Readmission”[Mesh] OR “Length of Stay”[Mesh]) AND (“Depression”[Mesh] OR “Depressive Disorder”[Mesh] OR “Bipolar Disorder”[Mesh] OR “Mood Disorders”[Mesh] OR “Depressive Disorder, Major”[Mesh]). The PsycINFO search used the following terms: (MM “Major Depression” OR MM “Bipolar Disorder”) AND (DE “Hospitalization” OR DE “Hospitalisation” OR DE “Hospital Admission”). No language restrictions were applied during the title and abstract screening; however during data extraction we further restricted our search to English papers alone. Titles, abstracts and full-text, if available, were screened by two separate investigators (Matthew Prina and Martijn Huisman) using the inclusion criteria reported below. Both investigators screened the entire sample of records, and reconciliation was carried out at two separate stages (during the title and abstract screening, and during the full text screening). During the full text screening, there was agreement between the investigators in 91% of the sample, and the remaining 9% of papers were jointly discussed before a final decision was made. The reference list of each paper was also scanned to identify further studies. Throughout this text we use the term depression, but this may be more accurately defined as depressed mood or depressive symptoms, given our population of interest. We did not restrict our selection of studies to exclude papers that assessed depressed mood or depressive symptoms short of actual clinical diagnoses of depression, as most of the identified studies focused on depressive symptoms.

### Inclusion and exclusion criteria

The following inclusion criteria were used to identify pertinent papers: a) Depression or depressive symptoms measured before admission; b) Hospitalisation in general hospitals including emergency departments; c) Papers including at least one of the following outcomes: hospitalisation, length of stay, or re-admission; d) The paper presents estimates of the association of depressive symptoms with at least one of these outcomes. The criterion ‘a’ was only applicable to studies that investigated the relationship of depressive symptoms with hospital admission, in order to exclude the possibility that depressive symptoms were subsequent to hospital admission (i.e. hospitalisation could be the cause of the depression). For the re-admission this criterion was not applied, as depressive symptoms would precede the outcome.

Papers were excluded if: a) they reported hospitalisation in mental institutions, rehabilitation clinics or nursing homes; b) the primary cause of admission was a mental disorder; c) the depressed individuals were not analysed as a separate category; d) they focused on bipolar disorders alone without specific mention of unipolar symptoms (i.e. the presence or absence of symptoms of mania was the exposure of interest); e) the study was not published in a peer-reviewed journal; f) the participants were not living in the community; g) the findings from the same study were already reported in another journal; and h) the study did not focus on community-dwelling individuals but solely on specific clinical populations (i.e. studies which focused on people already in hospital or solely on group of individuals with pre-existing conditions, such as asthma, stroke, and cancer).

### Data extraction

A standardised data extraction form was used to collate relevant information from each selected paper. The form included information on setting, sample size, participants' characteristics such as age and sex, depression scale used including cut-off point and time of assessment. Relevant outcomes were also identified and information regarding adjusted and unadjusted associations was extracted. References to other potential relevant studies were also gathered at the end of the extraction form.

### Quality

Elements of the design of identified studies were assessed by using an adapted version of the ‘quality assessment tool for quantitative studies’ [19]. The main components of this appraisal were: selection bias, study design and data collection methods, withdrawals and dropouts, type of analysis and confounders. Selection bias was assessed by extracting information of representativeness and response rate. The assessment of the study design was based on the sample size and by how depressive symptoms and outcomes were measured. For each quality metric, a score of one (‘weak’), two (‘moderate’) or three (‘strong’) was assigned.

### Data synthesis

A meta-analysis was only carried out for one outcome (hospital admission), as there was high heterogeneity in exposure and outcome measurements for length of stay and re-admission. A narrative synthesis of these outcomes, based on the ESRC guidance on the conduct of narrative synthesis in systematic reviews, was preferred [20].

For the main unadjusted outcome a fixed-effect meta-analysis was conducted and a pooled-estimate calculated, together with an I^2^ heterogeneity score [21]. A funnel-plot and a Galbraith plot based on the Egger test [22], that plots the standard normal deviate of the association effect against its precision, were used to assess biases in this analysis. In order to assess whether the pooled estimate was biased by the effect of any particular study we also carried out a sensitivity analysis by removing one study at the time and recalculating the pooled estimate. All the other results were synthesised in tabular format and described in the text. Three papers [23–25] did not report unadjusted associations and the authors were contacted for further details. However, data were not available from the corresponding authors.

## Results

**Fig. 1:**
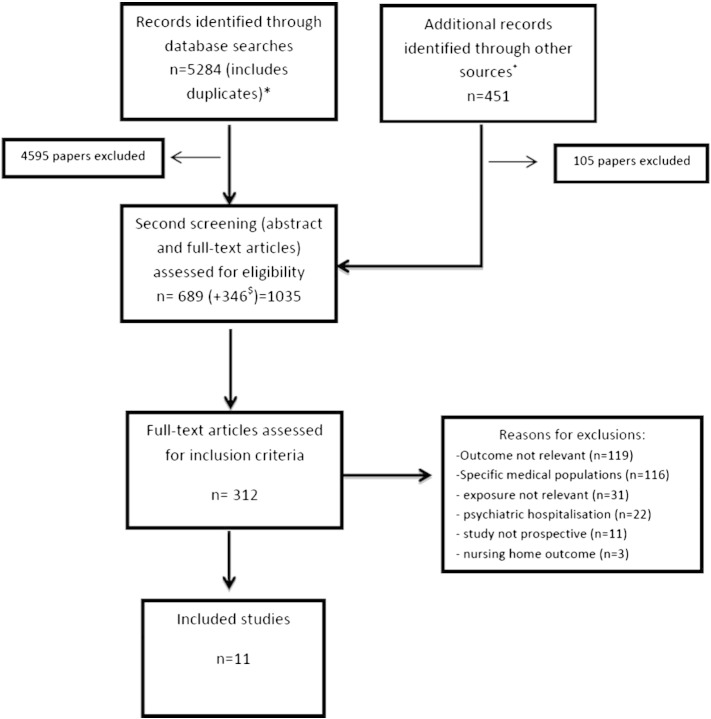
Study selection process. *Includes animal studies and studies relating only to psychiatric hospitalisation. + Includes 367 from updated searches and 84 extra references from other papers. $ studies identified from additional searches.

Of the potential 5284 papers identified through the database searches and the 451 that were recognised via other sources (e.g. on-line reports, reference lists, update searches), 11 were shortlisted. Fig. 1 shows the paper selection process, 82% of the papers were discarded directly from reading the title and abstract, most of which included studies relating solely to psychiatric hospitalisations. The eligibility of 1035 papers was assessed, and full text assessment was needed for 312 papers. Only 11 studies were carried out in the general population and fulfilled the other criteria. The two main reasons for exclusion were not reporting relevant outcomes (38%) or focusing on people with underlying cardiovascular, respiratory, diabetes and cognitive problems (37%).

The summary characteristics of these studies are reported in Table 1. Most studies were carried out in different countries and only the United States of America and the Netherlands were represented in two different studies. There were a total of 33,991 individuals, with the majority of studies focusing on older age groups. One exception was the study carried out by Koopmans and colleagues [25] which included individuals aged between 15 and 90. The prevalence of depressive symptoms varied significantly across the different studies, from 4.5% in a Singaporean study to 22.4% [26] in the study by Bula et al. [27]. This was partly due to the different cut-off points used and the different measurement scales. Most of the studies measured depression symptomatology using either the Geriatric Depression Scale (GDS) or the Centre for Epidemiological Study-Depression scale (CES-D). Follow-up times were also different across the studies with a range going from three months to 60 months. Ten studies investigated ‘hospitalisation’, four ‘length of stay’, and three ‘re-admission’. The quality of the studies was moderate to high. Only one study was deemed to be using an assessment for depressive symptoms that was too non-specific [25], which was based on asking the respondent whether they had any complaints of stress, depression or serious nervousness in the previous five years. Outcomes on the whole were well measured, with the exception of one paper [28], which asked the participants retrospectively whether they had been admitted to hospital in the year before interview. This measure was considered to be highly prone to recall bias. A systematic review of the accuracy of self-report of hospitalisation identified common issues with these measurements: — underreporting of visits, — over-reporting of visits in highly distressed individuals, — misclassification of visits in older and cognitively impaired individuals [29]. Most of the studies did not record withdrawals during the studies, with only two exceptions [28,30], primarily because the outcomes were tracked using databases, and the participants were not contacted a second time. Adjustment for potential confounders, in particular age, physical limitations and co-morbid illnesses, varied across the studies and was the factor that affected the quality appraisal scores the most, with two studies carrying out poor adjustment [25,31], and one only moderate [27]. The two studies with poor adjustment were only included in the unadjusted analysis that looked at depressive symptoms as a proxy for hospital admission. None of the studies included participants who had bipolar symptoms.

### Hospitalisation

**Fig. 2:**
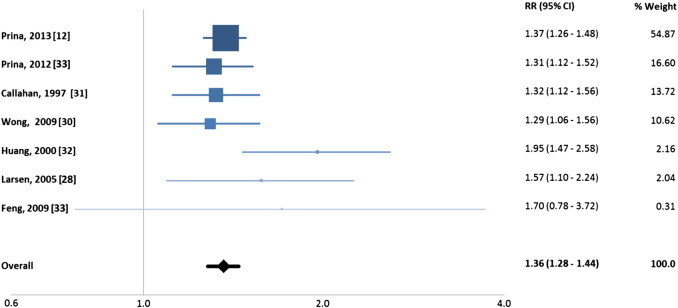
Fixed meta-analysis with pooled estimate of studies investigating the association of depressive symptoms with hospital admission in the general population.

Most studies reported a positive association between depressive symptoms and non-psychiatric hospitalisation before adjustment for potential confounders. Table 2 includes a list of unadjusted risk ratios calculated from the different studies, with an overall risk ratio for depressive symptoms of 1.36 (95% CI: 1.28–1.44) (Fig. 2). Unadjusted data were not available from the Koopmans, Laudisio and Rowan papers [23–25] and were therefore not included in the meta-analysis (Fig. 2). The pooled estimate was calculated using a fixed-effect model, given that no heterogeneity was measured by the I^2^ test (0.00%). Using a random-effect model did not affect the pooled-estimate (1.36, 95% CI: 1.28–1.44). The sensitivity analysis also indicated that the no individual study had a major effect on the pooled estimate. For example, removing Prina et al. [12] from the meta-analysis resulted in a pooled estimate of 1.35 (95% CI: 1.23–1.48), even though the weight of this study in the original meta-analysis was over 50%.

A funnel plot to investigate the association between standard errors and risk ratios was also drawn (supplementary Fig. 1) to see whether any underlying biases could be found in these associations. The funnel plot was symmetrical suggesting that publication bias may not play a role in this meta-analysis. Egger's test and related Galbraith plot (supplementary Fig. 2) were also used to investigate possible small-study reporting bias [22]. The p value was 0.24. No evidence of publication bias was detected but it is important to acknowledge that the power to detect such evidence in this setting was extremely low.

The role of potential confounders was investigated by most authors, with the exception of one study [31]. The degree of modelling varied across the studies, but most studies adjusted for co-morbidity and socio-demographics. The highest unadjusted relative risk (RR = 1.95,95% CI: 1.47–2.58) was reported by Huang and colleagues [32], for men and women combined. However, after full adjustment the association they reported remained statistically significant only among men aged 75 and over (RR = 3.43, 95% CI: 1.33–8.9). Of the other seven studies, four reported an independent effect of depressive symptoms on hospitalisation [12,23,24,30], whereas the other four [25,26,28,33] reported wide 95% CI confidence intervals after adjustment for potential confounders (Table 3).

Only one study [12] sub-divided depressive symptoms by increasing severity, reporting increasing hazard ratios for increasing scores on the geriatric depression scale. The adjusted HR for questionable depression (GDS scores: 1 to 4) were 1.70 (95% CI: 1.50–1.92), for mild to moderate depression (GDS scores: 5–9) 2.08 (95% CI: 1.68–2.58) and for severe depression 3.06 (2.10–4.46), compared with patients with no depression.

### Other outcomes

The other outcomes of interest that were reported in these studies are presented in Table 3. The associations reported in this table are for the fully adjusted models, and are therefore measuring the independent effect of depressive symptoms on these outcomes (i.e. independent of demographic factors and comorbidity or chronic disease severity).

Four studies investigated length of stay, and all reported a positive association between depressive symptoms and length of stay in hospital (Table 3). The duration of hospital admissions was measured differently in the different databases, hindering any further analysis on the data. Wong [30] grouped the number of days spent in hospital in zero, one to five, six to 15 and more than 16, whereas Rowan [24] used a dichotomous variable comparing individuals who spent more than three days in hospital with those who spent less than three. The two papers from Prina and colleagues [33,12] used mean and median length of stay to explore the relationship. The RR of increased duration of admission for individuals with depression ranged from 1.1 to 1.9 (Table 3).

Re-admission was reported in three studies. One used a dichotomous approach (re-admitted vs non re-admitted) and the other two used a continuous variable recording the number of hospitalisations. All the three studies reported positive associations in their multivariate models (Table 3). Bula et al. [27] reported the highest increased risk of hospital re-admission (adjusted HR = 1.50, 95% CI: 1.03–2.17), and both Prina [12] and Wong [30] found that depressive symptoms were associated with increased number of re-hospitalisations. A further study that our group carried out in the Dutch population [33] did not find any significant differences in the number of hospitalisations after adjustment for confounding variables (adjusted HR = 1.02, 95% C: 0.84–1.23).

## Discussion

The purpose of this paper was to give a comprehensive review of the literature regarding the association between depressive symptoms, non-psychiatric hospitalisations and related outcomes. Most studies of the relationship between hospitalisation and depressive symptoms have focused on specific clinical populations and were therefore excluded from this synthesis, whereas only a small percentage of studies was carried out in the general population of adults living in the community. Most of these latter studies reported an association between depressive symptoms, hospital admission, length of stay and number of re-admissions. Meta-analysis revealed depressive symptoms to be a potential indicator of subsequent hospitalisation RR = 1.36 (95% CI: 1.28–1.44), but the literature reported mixed findings on this relationship after adjustment for potential confounders. Depressive mood was also reported to be independently associated with longer length of stay with risk ratios varying from 1.1 to 1.9, and re-admission (lowest RR = 1.02, highest RR = 1.5).

### Limitations

Although it is possible that some papers were missed using the search strategy of this study, checking the reference list of each paper will have enhanced the sensitivity of our search. Therefore we believe that this possibility of missing relevant papers has been mitigated as far as possible.

The search was not originally limited to papers written in English. However, due to problems with accessibility and translation, one Japanese paper was excluded in the final selection screening [34]. We were therefore unable to assess whether this paper was relevant.

The meta-analysis only included seven (out of ten) studies that assessed depressive symptoms and hospitalisation. Although no biases were found using the Egger method, the power needed to detect any bias was probably low, given the small number of studies.

Several studies of patients in hospital were excluded because they did not identify depressive symptoms before admission [13,35–40]. This choice was dictated by wanting to analyse a prospective relationship, and not depressive symptoms driven by the admission itself. However, we cannot completely rule out the possibility that existing depressive symptoms in a number of individuals were the results of prior hospitalisations that were not recorded at the beginning of these studies. It has to be acknowledged that the inverse relationship (i.e. admission driving depression) is also an important one that needs to be further assessed by future studies.

All the studies measured depressive symptoms rather than major depressive disorder, and did not try to fully assess the severity of depressive symptoms. The risk to individuals suffering with major depressive disorder is probably higher than the risk carried by elevated scores on symptom rating scales such as the CES-D and GDS. One study [12] showed that the hazard ratios for hospitalisation were higher in patients with more severe depression, suggesting that the associations found in the literature are likely to be underestimates of the real link between major depression and medical admission. Using different scales for depressive symptoms, such as GDS, CES-D or BDI-II may also affect the risk estimates for hospitalisation. However, we could not see any significant differences in estimates between studies that used the GDS versus those that used the CES-D.

Finally, we were unable to differentiate between emergency admissions and inpatient hospital admissions, as the majority of studies did not report separate figures for diverse types of hospital admission. However, we are confident that all the events severe enough to require hospitalisation would have been captured regardless of whether the initial admission was in an emergency department.

### Interpretation of findings

Most studies in the general population reported a positive association between depressive symptoms and hospital admission, indicating that depressive symptoms are a risk indicator for future hospital admissions. These associations remained statistically significant in only half of the studies. The studies that did not find statistically significant associations were characterised by several potential limitations. Koopmans et al. [25] used a partially validated [41] assessment for depression, which measured self-reported depressive complaints, whereas Larsen [28] used an extremely long follow-up time of 5 years. Finally, it is possible that in our own previous study [33] we over-adjusted our statistical analysis. In this paper, a separate model was fitted for each factor, and it was found that adjusting for functional limitations consistently had the biggest impact on odds ratios. It is also worth noting that age and gender are likely to be true confounders, whereas functional limitations may be a mediator of this association. Unfortunately this hypothesis was not tested in any of the papers. Positive associations after adjustment for confounders were reported for both length of stay and for re-admission in all the studies, but were not heterogeneous for hospital admission. Most studies were conducted among older people. Physical illness and disability are the two of the most important risk factors for late life depression and older depressed people without physical illness or disability are a small minority [42]. Any attempt to disentangle the effects of depression and physical illness/disability in a naturalistic study is likely to fail because both depressive symptoms and physical illnesses are likely to be chronic or intermittent. This further hinders drawing a clear line between their respective impact in causing hospital admission. If it is true that depressive symptoms do not increase medical hospitalisation in the absence of medical co-morbidity, adjusting for physical comorbidity becomes important, as it suggests that any association of depression with hospital admission is due to underlying medical conditions. However, it is also possible that depressive symptoms may exacerbate medical symptoms or increase use of primary care services, and thus increase the risk of hospitalisation in people with existing medical conditions.

One of the major problems in comparing the studies was the highly variable follow-up periods. This could have affected the associations, as shown by Prina et al. [33], where the magnitude of the associations varied when different follow-up times were used. This is a common problem in longitudinal research on depression. Most of these studies only measured depressive symptoms at baseline, and never again during the course of the study. The average duration of an untreated episode of depression has been described to be around six to eight months with almost 50% of individuals relapsing within a 5 year period [43]. It is difficult to say whether misclassification bias could play a role in all of these studies as the episodic nature of depressive symptoms could have been resolved before hospitalisation, but it is likely to have played a stronger role in those studies that had a longer follow-up, in particular without having information on concurrent treatment and medication use. With longer follow-ups, we expect the effect of depression at baseline to become diluted, and with thorough treatment of depressive symptoms, the effect of depression should be ameliorated over time. This observation is somewhat supported by this review, which reported a neutral association by the study [28] with the longest follow-up time. Seven studies had a follow-up period above 12 months, suggesting that the overall estimate could be biased towards the null.

The relationship between hospitalisation and depressive symptoms could be explained by poor treatment adherence that is common in people suffering with mood disorders and that could translate into worsening of symptoms and hospitalisation for an underlying physical disorder [9,44]. Poorer clinical prognoses at admission, partly driven by the direct influence of depressive symptoms on the hypothalamic–pituitary–adrenal axis, the immune system, and the sympathetic nervous system, could also explain longer length of stays and risk of re-admission in people with depression [45]. Effective communication with health professional could also be affected in people with depressed mood. This could lead to a delay in a diagnosis and treatment [12]. Depressive symptoms may also impair a person's motivation towards recovery and their response to rehabilitation.

### Future implications

This review suggests that there is a link between depressive symptoms, hospitalisation and hospital-related outcomes. It is likely that this association is mediated by other factors and that depressive symptoms are not the sole independent factor in this relationship, even though most studies reported positive associations after adjustment for potential confounders. The majority of the studies we identified were conducted in older adults, who often have physical co-morbidities, and we believe it would be important to carry out more studies in younger patients with fewer somatic diseases in order to disentangle the bidirectional association between depression and physical co-morbidities. What transpires from this review, however, is that depression is a good predictive proxy for hospital outcomes. Further research with larger sample sizes, better assessment of depression (i.e. full clinical interviews and severity) and outcomes is needed, if potential pathways explaining this association are to be described in greater detail. Ideally this should be from a population-based sample rather than a clinic based one, to avoid the further biases of the latter. Insight can be gained from large-scale observation studies with rigorous assessment of depression and careful selection of confounders, which present results of unadjusted, partially adjusted and fully adjusted models. This unfortunately would require a large amount of resources. We advocate for the use of underutilised available resources, including linkage of cohort studies with clinical data. A number of health systems across the world collect routine clinical data, but these are often difficult or impossible to access due to strict regulations. It is important that these resources are made available to the wider scientific community, while respecting ethical and privacy norms. Moreover it is important to look at what happens to the level of hospital admission in experimental designs, collating data from RCTs and prospective follow-up patients with regard to their admission. In countries with good registration bases, this should be possible. Following such studies it might be possible to identify particular groups at higher risk and investigate whether targeted interventions in multiple health domains reduce these possible adverse consequences. Some encouraging results have been presented by Lin and colleagues [46], who showed that by improving depression care in a group of older adults with arthritis, functional outcomes and pain were also improved. Another trial showed that mortality risk in depressed patients could be lowered using a depression care management intervention [47]. It is not known whether this could be translated and used for reducing hospital admissions and length of stay, but it should be explored as a potential avenue of reducing hospital admissions, lengths of stay and, in the longer term, healthcare costs.

## Conflict of interest

The authors have no competing interests to report.

## Figures and Tables

**Table 1:** Summary characteristics of included studies investigating depression and hospitalisation in the community. dep = depressive symptoms, nodep = no depressive symptoms, GDS = Geriatric Depression Scale, CES-D = Center for Epidemiologic Studies Depression Scale Revised.

Authors, year	Country and sample size	Disease focus	Age	Depression measure	Depression prevalence	Follow-up time	Outcomes			Control for other variables
							Hospitalisation	Length of stay	Re-admission	
Bula et al., 2001 [27]	Switzerland n = 401	General but with previous hospitalisation	75 +	GDS	22.4%	6 months			x	Age, gender, living alone, education, income, previous admission, co-morbidities, functional limitations.
Callahan et al., 1997 [31]	USA n = 3767	General population	Mean: dep 66.6 nodep 68.1	CES-D	16.2%	12 months	x			n/a
Feng et al., 2009 [26]	Singapore n = 973	General population	55 +	GDS	4.9%	12 months	x			Age, gender, education, ethnicity, housing type, social-emotional support, co-morbidities, self-rated physical health, disability, cognitive impairment.
Huang et al., 2000 [32]	USA n = 3486	General population	65 +	CES-D	8.9%	6 months	x			Age, gender, marital status, income, level of education, urbanicity, co-morbidities, cognitive functions, number of medications
Koopmans et al., 2006 [25]	Netherlands n = 8698	General but with previous hospitalisation	Range: 15–90	Self-report	12.2%	12 months	x			Age, gender, living alone, education, marital status, co-morbidities
Larsen et al., 2006 [28]	Denmark n = 406	General population	Mean: 75	CES-D	16% men 30% women	60 months	x			Gender, living alone, self-rated health, previous hospitalisation
Laudisio et al., 2010 [23]	Italy n = 344	General population	Mean: dep 80 nodep 79	GDS	51%	12 months	x			Age, gender, previous hospitalisation, white blood cell count, medications
Prina et al., 2012 [33]	Netherlands n = 3304	General population	55 +	CES-D	16%	24 months	x	x	x	Age, gender, education, co-morbidities, functional limitations, smoking, alcohol problems
Prina et al. 2013 [12]	Australia n = 5411	General population	Mean: 78.6	GDS	6.3%	24 months	x	x	x	Age, education level, social support,
Rowan et al. 2002 [24]	Canada n = 3227	General population	Mean: dep 45 nodep 48	CES-D	n/a	12 months	x	x		co-morbidities, smoking
Wong et al. 2009 [30]	China n = 3770	General population	65 +	GDS	9.9%	n/a	x	x	x	Age, gender, disease severity, co-morbidities

**Table 2:** Absolute numbers of people hospitalised according to their depression status, and Risk Ratio (RR) with 95% Confidence Interval (CI). ^1^ Numbers based on overnight admissions alone.

	Depressed		Non-depressed		RR	95% CI
	Hospitalised	Not hospitalised	Hospitalised	Not hospitalised		
*Callahan*[31]	137	475	536	2619	1.32	(1.12–1.56)
*Feng*[26]	6	42	68	857	1.70	(0.78–3.72)
*Huang*[32]	n/a	n/a	n/a	n/a	1.95	(1.47–2.58)
*Koopmans*[25]	n/a	n/a	n/a	n/a	n/a	n/a
*Larsen*[28]	32	65	65	244	1.57	(1.10–2.24)
*Laudisio*[23]	n/a	n/a	n/a	n/a	n/a	n/a
*Prina*, *2012*[33]	177	1131	722	6252	1.31	(1.12–1.52)
*Prina*, *2013*[12]	197	86	2054	1975	1.37	(1.26–1.48)
*Rowan*[24]	n/a	n/a	n/a	n/a	n/a	n/a
*Wong*[30]	81	171	681	2049	1.29	(1.06–1.56)

**Table 3:** Outcomes in the general population. All the associations reported are adjusted. The exact adjustments are reported in Table 1. ^1^Coefficient from a binomial regression analysis. ^2^ Data presented only adjusted for age and gender, the full analysis was only reported after stratification by age and gender. ^3^ Adjusted analyses only available for any admissions (and not for overnight admission alone).

	Hospitalisation		Length of stay		Re-admission	
	Follow-up time	Direction of association	Measurement	Direction of association	Measurement	Direction of association
*Bula*[27]	–	–	–	–	Dichotomous	*Positive* HR = 1.5 (95% CI: 1.0–2.2)
*Feng*[26]	12 months	*Neutral* HR = 1.06 (95% CI: 0.44–2.58)				
*Huang*[32]	6 months	*Positive* RR^1^ = 2.25 (95% CI: 1.50–3.40)				
*Koopmans*[25]	12 months	*Neutral* Β^2^ = 0.022 (p > 0.05)	–	–	–	–
*Larsen*[28]	60 months	*Neutral* OR = 1.3 (95% CI: 0.5–3.3)	–	–	–	–
*Laudisio*[23]	12 months	*Positive* RR = 1.05 (95% CI: 1.01–1.09)				
*Prina*-*2012*[33]	12 months	*Neutral* OR = 1.01 (95% CI: 0.83–1.22)	Mean total length of stay (continuous)	*Positive* OR = 1.3 (95% CI: 1.2–1.5)	Number of hospitalisations (continuous)	*Neutral* OR = 1.02 (95% CI: 0.84–1.23)
*Prina*-*2013*[12]	24 months	*Positive* HR = 1.67^3^ (95% CI: 1.38–2.01)	Mean and total length of stay (continuous)	*Positive* RR = 1.9 (95% CI: 1.6–2.2)	Number of hospitalisations (continuous)	*Positive* RR = 1.5 (95% CI: 1.3–1.7)
*Rowan*[24]	12 months	*Positive* OR = 1.5 (95% CI: 1.1–2.0)	Number of days in hospital (dichotomous)	*Positive* OR = 1.8 (95% CI: 1.1–3.0)	–	–
*Wong*[30]	–	–	Number of days in hospital (categorical)	*Positive* RR = 1.1 (95% CI: 1.0–1.2)	Number of hospitalisations (categorical)	*Positive* RR = 1.3 (95% CI: 1.1–1.4)
