# Supplementary material for: The association between depressive symptoms in the community, non-psychiatric hospital admission and hospital outcomes: A systematic review
Source: J Psychosom Res. 2015 Jan;78(1):25–33. doi: 10.1016/j.jpsychores.2014.11.002 (PMC4292984; doi:10.1016/j.jpsychores.2014.11.002)

Supplementary figure 1 – Funnel plot with pseudo 95% CI of the included

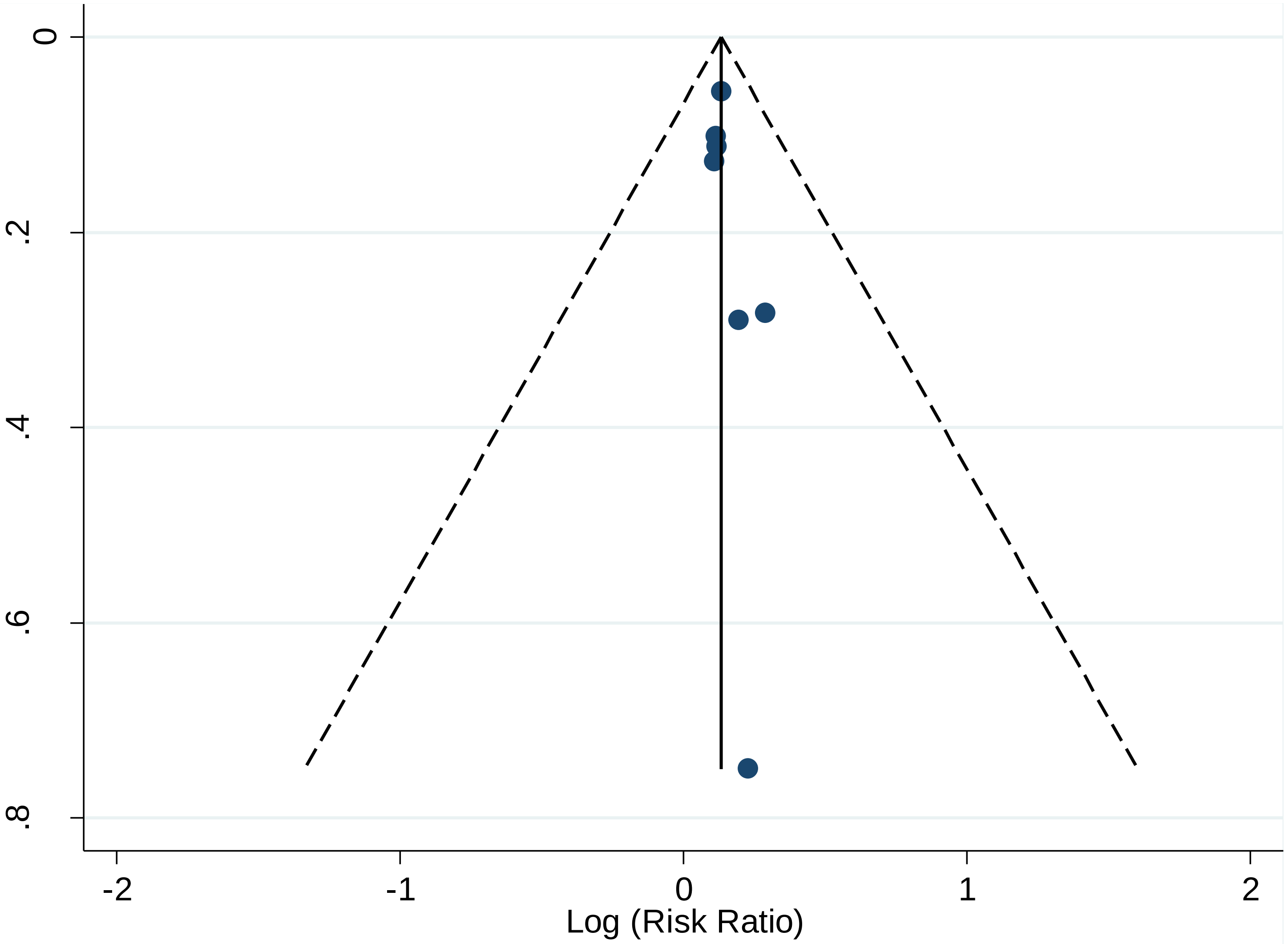

studies.

Supplementary figure 2 – Galbraith plot for the Egger test.

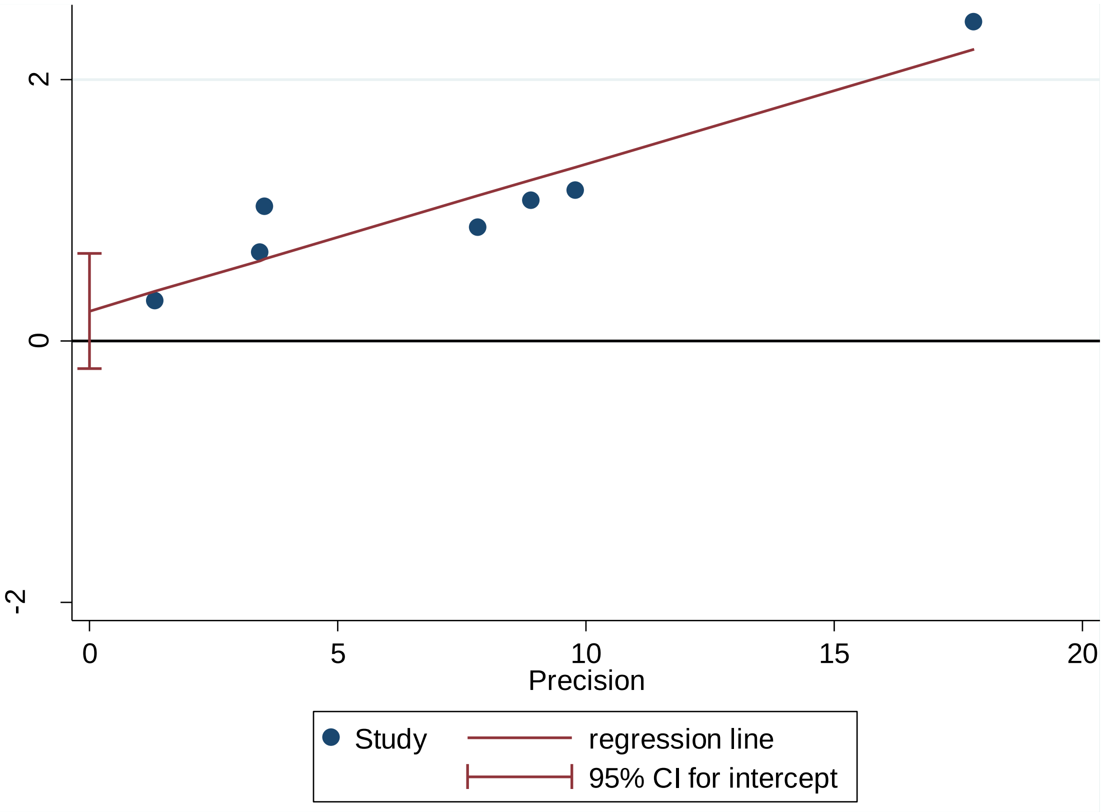

Supplement: Supplementary file 1 — Supplementary figures. [file mmc1.pdf]
