# Supplementary material for: The association between depressive symptoms in the community, non-psychiatric hospital admission and hospital outcomes: A systematic review
Source: J Psychosom Res. 2015 Jan;78(1):25–33. doi: 10.1016/j.jpsychores.2014.11.002 (PMC4292984; doi:10.1016/j.jpsychores.2014.11.002)
Supplement: Supplementary file 2 — Supplementary material. [file mmc2.doc]

**MOOSE Checklist**

**The association between depression in the community, hospital admission and hospital outcomes: a systematic review.**

A. Matthew Prina,

Institute of Public Health, University of Cambridge, UK

Centre for Global Mental Health, Institute of Psychiatry at King’s College London, UK

Theodore D. Cosco,

Institute of Public Health, University of Cambridge, UK

Tom Dening

Division of Psychiatry, University of Nottingham, UK

Aartjan Beekman

Department of Psychiatry, VU University Medical Center, Netherlands

Carol Brayne

Institute of Public Health, University of Cambridge, UK

Martijn Huisman

Department of Epidemiology & Biostatistics, EMGO Institute for Health and Care Research, VU University Medical Center, Netherlands

Department of Sociology, VU University, Netherlands

Corresponding Author :

Matthew Prina

Centre for Global Mental Health, Institute of Psychiatry at King’s College London, UK

E-mail : matthew.prina@kcl.ac.uk

| **Criteria** | | **Brief description of how the criteria were handled in the meta-analysis** |
| --- | --- | --- |
| **Reporting of background should include** | |  |
| √ | Problem definition | Depression is often seen in patients admitted to hospital, but the evidence of a potential link between depression and hospital admission has not been synthesised yet. |
| √ | Hypothesis statement | Depression increases the risk of hospital admissions and of worse hospital outcomes |
| √ | Description of study outcomes | Hospital admission  Length of Stay  Re-admission |
| √ | Type of exposure or intervention used | Depression or Depressive symptoms |
| √ | Type of study designs used | We included case-control studies, prospective cohort studies, and data-linkage studies. We excluded studies which investigated hospitalisation before depression. |
| √ | Study population | No restriction were placed |
| **Reporting of search strategy should include** | |  |
| √ | Qualifications of searchers | AM Prina – BSc (Hons), MPhil (Cantab) PhD (Cantab) (MRC Population Health Scientist Research Fellow)  TD Cosco – BSc, MSc (PhD Student)  Tom Dening - F.R.C.Psych, MD (Professor of Dementia Research)  Aartjan Beekman – MD, PhD (Professor of Psychiatry)  Carol Brayne – MD, PhD (Professor of Public Health Medicine)  Martijn Huisman – MSc, PhD (Associate Professor) |
| √ | Search strategy, including time period included in the synthesis and keywords | All searches were updated in July 2012. Details under search strategy. |
| √ | Databases and registries searched | Medline, Ovid SP, PsycINFO, Cochrane Collaboration Database. |
| √ | Search software used, name and version, including special features | We did not employ a search software. EndNote was used to merge retrieved citations and eliminate duplications. |
| √ | Use of hand searching | We hand-searched bibliographies of retrieved papers for additional references, |
| √ | List of citations located and those excluded, including justifications | The search process is described in the flow diagram (figure 1). The citation list is available upon request |
| √ | Method of addressing articles published in languages other than English | We limited papers to western Europeans languages. The eligibility of two papers wasn’t assessed because of this issue; |
| √ | Method of handling abstracts and unpublished studies | We had discussed this project at conferences, abstracts of conference proceedings were scanned and a few authors were contacted to identify further studies. |
| √ | Description of any contact with authors | Three authors were contacted to gain unadjusted data on their papers in order to include these studies in the meta-analysis. Data were not available from the corresponding authors. |
| **Reporting of methods should include** | |  |
| √ | Description of relevance or appropriateness of studies assembled for assessing the hypothesis to be tested | Detailed inclusion and exclusion criteria were described in the methods section. |
| √ | Rationale for the selection and coding of data | Data extracted from each of the studies were relevant to the population characteristics, study design, exposure, and outcome. |
| √ | Assessment of confounding | Adjustment in the analyses (if any) was reported in the summary characteristics of included studies (table 1). |
| √ | Assessment of study quality, including blinding of quality assessors; stratification or regression on possible predictors of study results | The quality of the studies was assessed by using an adapted version of the ‘quality assessment tool for quantitative studies^5^. Results are reported in the supplementary table 1. |
| √ | Assessment of heterogeneity | Heterogeneity of the studies was explored using I^2^ statistic that provides the relative amount of variance of the summary effect due to the between-study heterogeneity. Moreover for the other outcomes heterogeneity was explored narratively in the discussion section. |
| √ | Description of statistical methods in sufficient detail to be replicated | Description of methods of meta-analyses and of publication bias are detailed in the methods. |
| √ | Provision of appropriate tables and graphics | We included 1 table detailing summary characteristics of the studies, 2 tables to summarise outcomes, 1 flow chart, 1 forest plot for one outcome, 1 supplementary table with the quality of the studies and 2 supplementary tables to explore potential biases. |
| **Reporting of results should include** | |  |
| √ | Graph summarizing individual study estimates and overall estimate | Figure 2, table 2, table 3 |
| √ | Table giving descriptive information for each study included | Table 1 |
| √ | Results of sensitivity testing | Narrative format in the test |
| √ | Indication of statistical uncertainty of findings | 95% confidence intervals were presented with all summary estimates, or p values when the confidence interval wasn’t available. I^2^ was also reported in the forest plot. |
| **Reporting of discussion should include** | |  |
| √ | Quantitative assessment of bias | Biases were assessed graphically using standard ways of assessing biases (A funnel-plot and a Galbraith plot based on the Egger test). |
| √ | Justification for exclusion | We excluded studies that did not identify depression before or within the first 24 hours of admission. This choice was dictated by wanting to analyse a prospective relationship, and not depressive symptoms driven by the admission itself. |
| √ | Assessment of quality of included studies | We reported the results of the quality assessments, and this was further assessed in the limitations/interpretation of findings. |
| **Reporting of conclusions should include** | |  |
| √ | Consideration of alternative explanations for observed results | We discussed the effect of length of follow-up, and of other confounders on the associations, and provided potential explanations for this association. |
| √ | Generalization of the conclusions | Generalisability is discussed in the limitations and also in the interpretation of findings (most studies were conducted in the older population). |
| √ | Guidelines for future research | We recommend future studies large scale studies and in particular experimental designs to gain a better insight in this relationship |
| √ | Disclosure of funding source | No separate funding was necessary for the undertaking of this systematic review. The corresponding author was funded by the MRC. |
